# Organ Trafficking and Migration: A Bibliometric Analysis of an Untold Story

**DOI:** 10.3390/ijerph17093204

**Published:** 2020-05-05

**Authors:** Juan Gonzalez, Ignacio Garijo, Alfonso Sanchez

**Affiliations:** Department of International Relations, Universidad Loyola Andalucía, 41704 Dos Hermanas, Spain; jbgonzalezblanco@al.uloyola.es (J.G.); igarijocampos@al.uloyola.es (I.G.)

**Keywords:** organ trafficking, migration, health, human trafficking, THBOR, refugees

## Abstract

The debate over trafficking of human beings for the purpose of organ removal (THBOR) remains largely absent from policy debates, as its crime is hardly detected, reported and sparsely researched. However, criminal networks continue to exploit vulnerable populations, particularly migrants. To help bridge this gap in knowledge, we employ a bibliometric analysis to examine whether the nexus between organ removal and migration is being addressed by the current academic literature. Our results indicate that (1) research exploring the link between THBOR and migrants is relatively scarce; (2) organ trafficking literature output is largely clustered in a couple of Western countries, and (3) despite the international nature of the topic, most empirical studies on organ trafficking and migration lack representation within the social sciences and humanities. Taken together, our results point to a huge gap on scientific publications between THBOR and migration. Quantitative data is required to lift the current knowledge constraints and better inform policymakers.

## 1. Introduction

The trafficking of human beings for the purpose of organ removal (THBOR) is not a new phenomenon [1]. With a shortage of legally sourced organs around the world, it is estimated that the illegal trade of human organs generates about 1.5 billion dollars each year from roughly 12,000 illegal transplants [2]. THBOR has serious consequences for human security, particularly for the most vulnerable populations, such as the unemployed, homeless people and migrants. For instance, in 2017, a growing number of organ trafficking cases was uncovered in Lebanon, as Syrian refugees were desperate to support themselves and their families [3]. Since the Declaration of Istanbul on Organ Trafficking and Transplant Tourism, governments and non-governmental organizations (NGOs) have begun to provide rough estimates about THBOR, and yet, little academic attention has been devoted to the study of such a global phenomenon [4]. In 2004, the WHO urged governments to take measures against organ trafficking and protect those who are the most vulnerable. Such efforts culminated with the Declaration of Istanbul on Organ Trafficking and Transplant Tourism, where more than 150 scientific researchers from 78 different countries concluded that commercialized transplants, organ trafficking, and transplant tourism should be prohibited [4]. In this paper, we aim to shed light upon the current state of the literature on: (1) THBOR and (2) its relationship to migrants. We employ a bibliometric analysis with a global sample for the 1990–2019 period, to find the current trends and shortcomings in the literature.

In 2018, the International Labor Organization (ILO) asserted that about 40 million people were victims of Human Trafficking—roughly the population of Iraq today [5]. About 90 percent of all detected cases were for sexual exploitation or forced labour purposes [6]. The remaining 10 percent of cases are often lumped together in the “other forms” category—including organ removal. Organ trafficking is a broad concept that includes several illegal activities, of which the main goal is to profit from human organs and tissue, for the sole purpose of transplantation. These activities include THBOR, transplant tourism and trafficking in organs and tissues [7,8,9]. Despite international and domestic efforts, about 10 percent of all transplants worldwide are believed to be illegal—approximately 12,000 organs per year [10]. While the number of reports on victims of trafficking in people has increased, only 700 victims of THBOR were detected from 25 countries for the 2006–2019 period [6]. 

Migrants are amongst one of the most vulnerable populations for organ trafficking [11,12]. Many migrants often face poor socio-economic and political conditions in their own countries. However, situations of vulnerability can occur while en route or in host areas, as migrants are exposed to abuse and exploitation by smugglers and opportunists [11]. For instance, in 2015, Pakistani refugees in Lebanon escaping the Syrian civil war were prevented from re-registering as refugees in a second country by the UN. In the words of an organ broker, “those who are not registered as refugees are struggling. What can they do? They are desperate and they have no other means to survive but to sell their organs” [11]. Moreover, a migrant’s individual vulnerability can increase due to personal circumstances. Such situations arise when migrants travel with children, elderly, people with disabilities and the like [13]. The previously mentioned organ broker also acknowledged that one of his clients was a 17-year-old boy who left Syria after his father and brothers were killed. He had been in Lebanon for three years with no work and mounting debt, struggling to support his mother and five sisters. “He agreed to sell his right kidney for US$ 8000” [11]. 

To summarize, THBOR is a global problem with health and human rights consequences for millions of people, especially for those in vulnerable situations, such as migrants. The following research questions are relevant to our study: To what extent is scientific research addressing THBOR? Are migrants included in this research? Where is most of the published output from THBOR and migrants being produced?

## 2. Materials and Methods 

### 2.1. Study Design 

We employed a bibliometric analysis to accomplish the goals of our study. A bibliometric analysis consists of the application of quantitative analysis techniques to data concerning scientific production, such as journal articles and their accompanying citation counts [14,15]. It is ideal to quantitatively measure the trends, volume, and scope of publications on THBOR. The use of a bibliometric analysis also allows us to find the geographical distribution of knowledge production about THBOR and migrants, as well as to explore the most frequently used methodological approaches in the study of organ trafficking. Therefore, bibliometric analysis constitutes a useful tool to quantitatively explore the evolution and situation of THBOR literature and particularly to find if, and to what extent, migrants, as one of the most vulnerable groups, are being included in publications dealing with such a relevant topic. In conclusion, it provides useful information for researchers and professionals to evaluate publication activity, offering a basis for future research.

### 2.2. Search Strategy

Table 1 presents our search strategy: iterations, keywords, and exclusions. Keyword selection is of great importance in bibliometric analysis, as keywords searched will determine the documents retrieved and, therefore, the findings and results. Search keywords were selected from previous published literature, relevant WHO documents such as resolution 44.25 [16], the WHO Global Glossary of Terms and Definitions on Donation and Transplantation [17], and the Declaration of Istanbul [4]. The second column in Table 1 presents the keywords searched among the topic field tags in Web of Science (WoS). Topic field tags include titles, abstracts, keywords and descriptors of every document. Although the search was not restricted to any language, most of the results come from English language publications. Iterations with specific organs were also used, as some publications do not address THBOR as a general topic, but focus on the trafficking or illegal transplantations of a specific organ or tissue. The specific organs utilized in our search are presented in the first column of Table 1. These keywords were selected for iterations because they are estimated to be the most frequently illegally removed [2]. Finally, excluded keywords were selected to minimize the number of false-positive results. As a result of this iterated search, we obtain a database of 482 publications, which will be used for the following bibliometric analysis. 

### 2.3. Source of Information

Data were obtained from the Web of Science (WoS) database for the 1990–2019 period—no publications relating to organ trafficking were found prior to 1990. WoS is a global citation search engine whose database concentrates “ideas across disciplines and time from over 1.7 billion cited references from over 159 million records” from leading academic, corporate, and government institutions over 115 years [18]. WoS includes the Science Citation Index Expanded (SCIE), Social Sciences Citation Index (SSCI), Arts and Humanities Citation Index (AHCI), Emerging Sources Citation Index (ESCI), Book Citation Index (BKCI), Conference Proceedings Citation Index (CPCI), and Current Chemical Reactions and Index Chemicus [19,20,21,22,23,24,25,26]. The database was created by Clarivate Analytics, an international, well-known enterprise dedicated to innovation and the delivery of critical data and information for, among others, scientific and academic research [27]. Furthermore, as Web of Science has been indexing every piece of content, and supplies a great variety of filtering options, it is the most reliable database for bibliometric analysis. 

### 2.4. Bibliometric Indicators

Bibliometric indicators measure knowledge production about a certain topic (e.g., THBOR). The number of publications and citations are the most useful and widely employed metrics, as they quantitatively measure the volume of research output, and the impact and influence of it, respectively. Our bibliometric indicators are presented as a time-series to show a trend in THBOR research. We are particularly interested in the most productive journals and institutions, as well as their country of origin. This will allow us to analyse the spatial distribution of global output. In line with previous bibliometric analysis literature, we utilize a threshold of ten when presenting our results [28,29,30,31]. These indicators, namely the number of publications and citations, can be employed to further analyse research trends on the topic of THBOR, with the focus on which research approaches are being used, especially on how migrants’ health has been included in organ trafficking research. 

### 2.5. Research Approach and Specificity

The Web of Science categories show which research field a publication belongs to and which methodological approach was utilized. Bibliometric indicators are used to show the most common fields of study conducting research on THBOR. Previous studies on human trafficking have found health-related approaches to be underrepresented in the literature [25]. However, THBOR itself is a health-related issue. Therefore, we cluster all health-related publications for the following research areas (Web of Science categories): biomedical social sciences, cardiovascular system and cardiology, cell biology, gastroenterology and hepatology, general and internal medicine, genetics and heredity, health care sciences and services, haematology, immunology, infectious diseases, legal medicine, medical ethics, microbiology, neurosciences and neurology, nursing, obstetrics and gynaecology, pathology, pediatrics, pharmacology and pharmacy, psychiatry, psychology, reproductive biology, research and experimental medicine, respiratory system, surgery, transplantation, urology and nephrology. Publications that did not fall under these fields of study were classified as non-health related.

An additional analysis of the database metrics was conducted to classify publications depending on their specificity. Some publications focus their THBOR research exclusively on the removal of a specific organ(s) or address the different dynamics between trafficking of different organs. Organ-specific documents are defined as those making the explicit mention of any organ in their title, abstract or keywords (see Table 1 for specific organs utilized in our search). We believe that this specificity will allow us to shed light on what kind of specific organs have researchers devoted most of their attention to, as well as the spatial distribution of where these research specific clusters are taking place across the world. Publications that mention a specific organ are henceforth referred to as “organ-specific publications.” 

Given that one of the main purposes of our study is to analyse THBOR research that addresses migrants, publications were classified depending on their inclusion of migrants. We assume that when migrants are mentioned in the title or abstract of a document, it means that the article takes into account these relevant groups, as they may be the main research objects. Publications studying migrants are defined as those including the following words in the title or abstract: migrant/s, emigrant/s, immigrant/s, migration, emigration, immigration, refugee/s, asylum seeker. 

## 3. Results

Our results suggest four key findings. First, a lack of research on organ trafficking. Second, the research that addresses the link between THBOR and migrants is staggeringly scarce. Third, most organ trafficking research originates from Western countries. Finally, the bulk of the literature comes from the medical sciences and there is a wide gap within the fields of social science and the humanities.

### 3.1. Volume of Publications: Temporal Analysis

**Figure 1:**
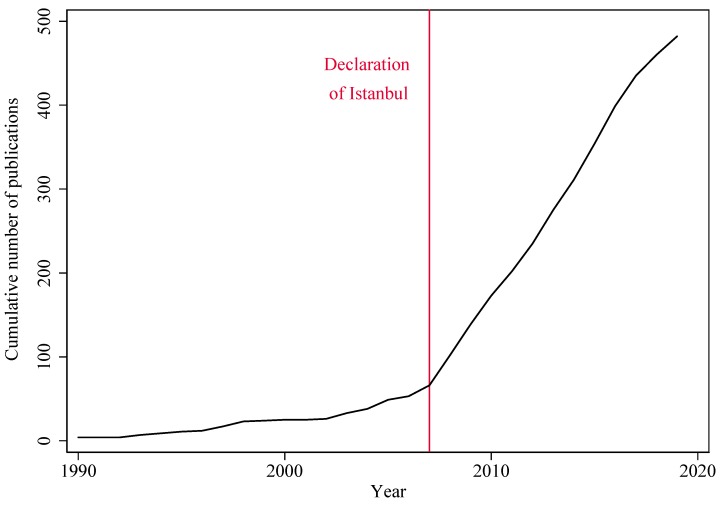
Total number of publications on THBOR per year.

In total, 482 documents about organ trafficking were used in our analysis. Figure 1 shows an increase in the total number of publications pertaining to organ trafficking from 1990 to 2019. Researchers have increasingly paid attention to this issue since the Istanbul Summit and the following publication of the Declaration of Istanbul on Organ Trafficking and Transplant Tourism in 2008. On average, before the Declaration of Istanbul, about 3.7 organ-trafficking-related documents were published per year. After the Declaration, the average increased to 34.7 documents per year. Therefore, we conclude that international attention to organ trafficking has led to a boost in publications about THBOR.

The momentum created in 2008 can be also appreciated in the total number of citations received by organ trafficking research studies. Documents published in 2008 received more citations than those published in any other year and per publication (17.5) received double the citations than the overall average (8). The impact and influence of the Declaration of Istanbul of 2008 are clearly evident, but the momentum may have slightly decreased in the last couple of years, as suggested by the slight decrease in the slope of the curve in Figure 1. 

### 3.2. Spatial Distribution

The retrieved documents were published in 219 different journals. As Table 2 shows, the most active journal in the field of organ trafficking was the American Journal of Transplantation, with a total number of 45 publications, closely followed by Transplantation, with 41 publications. Almost one in every five articles (18%) on organ trafficking was published in these two journals. However, documents published in The Lancet (30.8) and Current Opinion in Organ Transplantation (21.6) received the highest number of citations per article. As can be inferred from the journals’ titles, most active journals belong to different medical research areas. 

A similar pattern unfolds when we look at authors’ institutional affiliations. Authors from the documents retrieved were affiliated to a total number of 859 institutions from 75 different countries. Table 2 also shows that Harvard University (52) and the University of California (51) are the leading institutions in THBOR research, when measured in terms of total publications. As was the case with most journals, the leading institutions on organ trafficking are concentrated in Anglo-Saxon countries: four of them in the USA, two of them in Australia, and one in Canada. 

**Figure 2:**
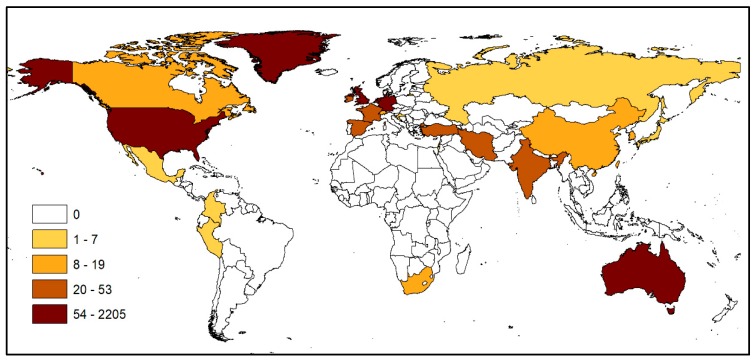
Total citations by journals’ countries of origin, 1990–2019.

Figure 2 displays the most influential countries in THBOR research when using the total number of cited papers as a proxy. The geographical distribution illustrates that most of the literature is shaped and produced by Western countries. First, 85% of all cited organ trafficking-related articles are concentrated in the USA and UK. Second, while the rest of the leading countries are not Anglo Saxon, they tend to be economically affluent Western democracies—with the exception of South Africa and Turkey [32]. Although such a trend is not unexpected, we find important to point out that countries in Latin America, Africa and Asia that have the highest number of THBOR victims are underrepresented within the current literature [2]. Ironically, some rich countries that are amongst the primary organ-importers are also shaping the discourse on THBOR, while poor countries—often transit and/or destination areas—are largely underrepresented [2,19,20,21]. 

### 3.3. Research Areas and Specific Organs

**Figure 3:**
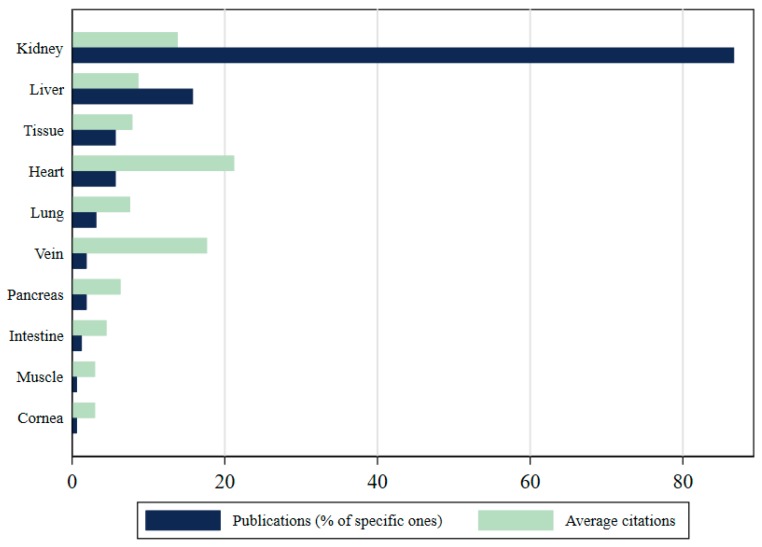
Most frequently studied organs in the trafficking of human beings for the purpose of organ removal (THBOR) research. Publications can simultaneously refer to more than one organ (percentages are out of 100).

Most of the publications in our sample address organ trafficking in broad terms and do not focus on the trafficking of a certain organ or tissue. Only 158 of all the organ trafficking studies retrieved (32.7%) mentioned a specific organ. Figure 3 illustrates the most common organs being addressed in the organ trafficking literature: almost 85% of the publications make references to kidneys, 16% mention liver, and about 6% refer to the heart. This ranking is consistent with the estimates of removals by organ, as most of the frequent illegal transplants involve kidney (67%), liver (22%) and heart (6%). Unexpectedly, publications that cover heart trafficking have a higher impact, receiving 21.2 citations per document, well above the average impact (eight citations per publication). 

**Figure 4:**
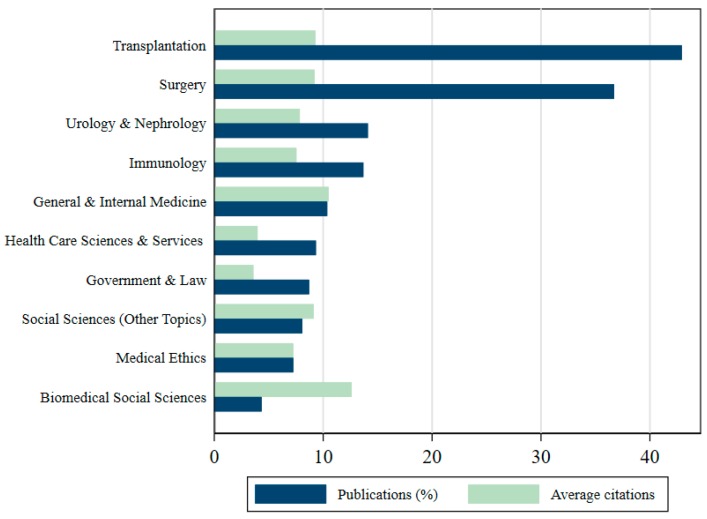
Top ten research areas in THBOR literature. Publications can simultaneously belong to more than one research area (percentages are out of 100).

When we consider that 76% of all THBOR research is done in the medical field, it comes as no surprise that eight of the top ten research areas within the THBOR literature belong to the medical field, as Figure 4 illustrates. This is a logical conclusion considering that organ removal/transplants require medical professionals. Nevertheless, it is surprising that such a transnational phenomenon and human rights issue is not being addressed by other fields such as international relations, which comprises only 2% of all publications. 

### 3.4. THBOR and Migration

**Figure 5:**
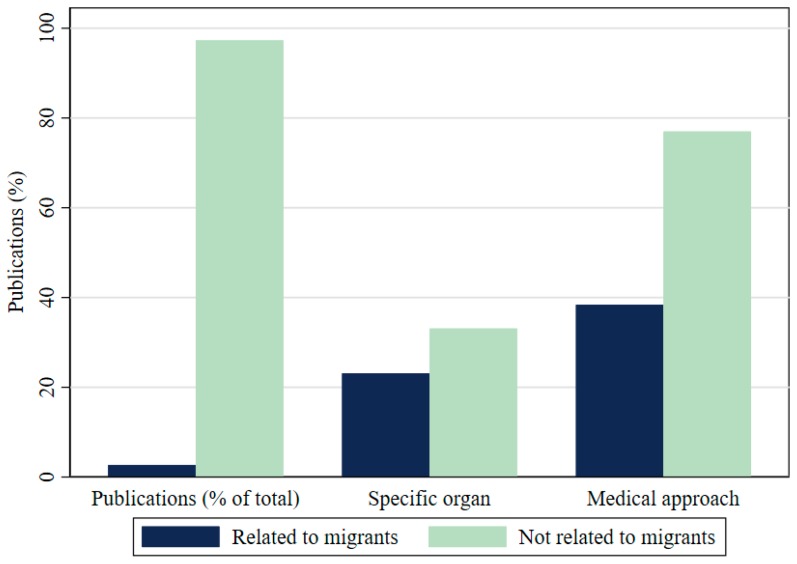
Migrant inclusion/exclusion in THBOR research.

Is THBOR research addressing migrants? We find that migrants are not receiving proper attention within our main sample: only 13 documents, or 2.7% of 482 publications, address migrants. The omission of migrants from publications can be clearly seen in Figure 5. Migrants are not only neglected from research, but when they are acknowledged in the literature, the research focus is quite different. Publications that address migrants are less organ-specific, with just 21% of them mentioning any specific organ. This is a third less than non-migrant-related documents. 

The gap becomes wider when we narrow our focus to examine how many of the 13 documents specifically address the health of migrants. Previously, we established that the medical field was the most prolific on THBOR literature—arguably due to its natural relationship to the field of organ transplantation. However, such prolific production abruptly drops when we include migrants in our search: out of all 482 publications retrieved about organ trafficking, only 5 specifically address migrants’ health. That is to say, about 1% of the entire THBOR literature has considered the relation between organ trafficking and migration from a medical field—a problem given that migrants are a particularly vulnerable group.

## 4. Discussion

The trafficking of human beings for the purpose of organ removal (THBOR) is a highly relevant health and human rights issue that disproportionately impacts migrants. The number of publications on THBOR has been on the rise since the Declaration of Istanbul on Organ Trafficking and Transplant Tourism of 2008, which puts the issue on the agenda of both International Organizations and researchers. However, the momentum created by the Declaration of Istanbul may have been slowing down in the last few years (as shown in Figure 1), and THBOR research has yet to catch up with the literature on other types of human trafficking. Despite the growth of scientific output on organ trafficking over the past decade, this literature comprises only 7% of the bulk of human trafficking literature [25]. 

Second, although THBOR disproportionately targets vulnerable populations such as migrants, the literature has largely excluded them from the issue. Only 2.7% of the entire THBOR literature in our sample acknowledges any direct or indirect relationship to migrants. This gap leaves the most vulnerable groups out of the academic and policy discourses, as well as the potential creation of any socio-political and healthcare policy agenda to tackle organ trafficking. Similarly, publications that do include migrants in their studies are less organ-specific, which is problematic, given that this is vital to understand the different dynamics of each organ being smuggled. 

This exclusion is even greater from the perspective of the migrant’s health. Despite THBOR being a health issue since it entails the need for surgery to remove the organs, most publications about migrants in the THBOR literature do not approach it from a medical field. The health of migrants is currently being neglected by scientists of all stripes. Future research should address this blind spot in the literature.

Such a limited number of publications can be attributed to a combination of factors. First, the differences in terms and definitions (before the Declaration of Istanbul and the WHO Glossary) make it difficult to conduct research consistently and in a homogenous manner. Additionally, the hidden and illegal nature of THBOR poses serious challenges to research on this topic, as it is complicated to gather reliable information—and often when the data is available, it is suppressed for political expediency. The lack of information is especially alarming in the case of quantitative data, imposing severe methodological constraints to organ trafficking research. Future research should further address THBOR in order to close the literature gap with other types of human trafficking research, focusing mostly on increasing reliable quantitative data on a global level. 

Given the global complexity of THBOR, it is surprising that in most areas of social science research (e.g., international relations), there is limited scientific knowledge pertaining to THBOR being generated. In the last 29 years, just 10 publications have examined organ trafficking from an international relations perspective. The phenomenon of organ trafficking and transplant tourism is a global problem, as trafficking networks are usually transnational—especially those targeting migrants—and often persist along the divide between rich (recipients) and poor (transit or origin) countries. Thus, research on THBOR should address this lack of attention from the angle of international relations and further analyse THBOR in the context of international migration dynamics. When mapping the country of origin of THBOR research, a remarkable bias is found. First, the skew highlights the presence of English-speaking countries, such as the USA, the UK, South Africa and Australia, which are among the most influential countries in terms of the cited work on this topic. Second, most of these countries are also net migration receiver countries. However, origin and transit countries for migration, which suffer more from THBOR, are not the ones publishing research on the topic (except Turkey and South Africa). To be able to address organ trafficking, research should also be promoted, incentivized and funded in developing and transit countries. Research made by these countries can best consider the local and regional dynamics of organ trafficking, as they are usually the victim’s country of origin or where the organ removal surgery is performed.

### Limitations of Study

While Web of Science is an internationally recognised group of databases which offers reliable information, it also has limitations. The main one is the language bias, which results in English-speaking countries being over-represented. This may explain why China, India, or Germany are not as present as they should be, regarding their number of publications. In addition, unlike many other bibliometric analyses, this paper does not include grey literature, as they are not included in the Web of Science. Thus, these limitations need to be considered when studying the findings of the investigation. Furthermore, the country of origin of the journal is not necessarily the same as the one of the editor or writers. Consequently, the results only show the countries in which more resources are being allocated. Moreover, we also acknowledge that it is possible that some of our retrieved literature could include the migrant population as well, but without mentioning this subgroup—perhaps due to legal/ethical constraints. Nonetheless, the available research so far does not allow for a robust examination of the impact of organ selling on migrants.

## 5. Conclusions

The current study is, to the best of our knowledge, the first to assess publication activity relating to the topic of trafficking of human beings for the purpose of organ removal. It has shown the huge quantitative impact the Declaration of Istanbul of 2008 had on stimulating research about organ trafficking. However, the literature on organ trafficking has yet to catch up with research on other typologies of human trafficking that are more established. We conclude that there is a clear need for improving and increasing quantitative data on THBOR, particularly focusing on migrants, who are usually the victims of organ trafficking and need to be included in the published articles. Currently, migrants are being ignored by research on THBOR, and medical research about organ trafficking and migrants is all but completely neglected. Furthermore, while academic publications mostly originate from rich countries, poor and middle-income countries are actually the most vulnerable, because they are either transit or origin areas for most international migrants. Future research should further address the relationship between migration, migrant’s health and organ trafficking, in order to develop efficient public policies against organ trafficking and transplant tourism. This can be done by incorporating and advancing scientific output by developing countries. 

## Figures and Tables

**Table 1:** Search strategy used in Web of Science (WoS) to retrieve data.

Iterations	Keywords	Exclusions
organ organs kidney*/renal liver heart*/cardi* lung*/pulmon* pancreas* intestine* tissue* ocular* cornea* vein*/vascular* skin* bone*	TS = (“transplant tourism” OR “transplant commercialism” OR “commercial transplantation” OR “organ traffic*” OR “illegal organ trade” OR “ illegal organ sale*” OR “commercially motivated organ transplant*” OR “paid organ transplant*” OR “illegal organ commerc*” OR “organ laundering” OR “illegal organ removal”)	NOT TS = (“cell*” OR “gene” OR “genetic” OR “transport” OR “chemist*” OR “biochemist*” OR “internet” OR “molecule*” OR “DDoS” OR “halogens” OR “hardware” OR “aerosol” OR “single-shell”) NOT PY = 2020

Note: symbol “*” includes all possible grammatical endings.

**Table 2:** Top ten most active journals and institutions on THBOR research.

Rank	Journal	*N*	Mean Citations	Institutions	*N*
1	American Journal of Transplantation (USA)	45	14.0	Harvard University (USA)	52
2	Transplantation (USA)	41	5.4	University of California (USA)	51
3	Transplantation Proceedings (USA)	19	9.4	Erasmus Univ. Rotterdam (Netherlands)	32
4	Kidney International (USA)	14	8.8	Massachusetts General Hospital (USA)	22
5	Transplant International (USA)	14	20.3	Tel Aviv University (Israel)	20
6	Clinical Transplantation (Denmark)	11	10.5	University of Minnesota (USA)	19
7	Lancet (UK)	11	30.8	University of Melbourne (Australia)	17
8	British Medical Journal (UK)	10	2.8	University of Sydney (Australia)	11
9	Current Opinion in Organ Transplantation (USA)	9	21.6	University of British Columbia (Canada)	10
10	Experimental and Clinical Transplantation (Turkey)	9	5.8	University of Cape Town (South Africa)	10

*N* is the total number of publications.
